# Biventricular dysfunction predicts mortality in ST elevation myocardial infarction patients with cardiogenic shock

**DOI:** 10.1186/s43044-024-00599-8

**Published:** 2025-01-08

**Authors:** Angga Dwi Prasetyo, Hendry Purnasidha Bagaswoto, Firandi Saputra, Erika Maharani, Budi Yuli Setianto

**Affiliations:** Department of Cardiology and Vascular Medicine, Faculty of Medicine, Public Health, and Nursing, Universitas Gadjah Mada/Dr. Sardjito General Hospital, Jalan Farmako Sekip Utara, Yogyakarta, 55281 Indonesia

**Keywords:** Echocardiography, ST elevated myocardial infarction, Cardiogenic shock, Mortality

## Abstract

**Background:**

The incidence of mortality in patients with cardiogenic shock due to ST elevation myocardial infarction (STEMI) remains high even with prompt reperfusion therapy. Ventricular systolic dysfunction is the primary condition causing cardiogenic shock in STEMI. Studies have been widely conducted on the left ventricle (LV) and right ventricle (RV) systolic dysfunction related to mortality events. However, the parameters of biventricular systolic dysfunction predicting mortality as a stronger predictor of mortality are still unclear. Accordingly, we evaluated the predictor mortality value of biventricular systolic dysfunction in STEMI patients with cardiogenic shocks. Based on The Society for Cardiovascular Angiography and Intervention classification, we analyzed data from November 2021 to September 2023 at Dr. Sardjito General Hospital in Yogyakarta, Indonesia, using the Sardjito Cardiovascular Intensive Care (SCIENCE) registry with a retrospective cohort design. Multivariate logistic regression analysis was used to assess predictors of in-hospital mortality.

**Results:**

There were 1,059 subjects with a mean ± SD age of 59 ± 11 years, dominated by men (80.5%) who met the inclusion and exclusion criteria. Based on multivariate analysis, biventricular dysfunction (BVD) is a factor that significantly increases the risk of in-hospital mortality (Odds ratio [OR], 1.771: 95% confidence interval [CI] 1.113–2.819; *p* = 0.016). Other significant factors affecting mortality were renal failure (OR, 5.122; 95% CI 3.233–8.116; *p* < 0.001), percutaneous coronary intervention (PCI) (OR, 0.493; 95% CI 0.248–0.981; *p* = 0.044), and inotropic/vasopressor (OR, 6.876; 95% CI 4.583–10.315; *p* < 0.001).

**Conclusions:**

Biventricular dysfunction significantly increases the risk of in-hospital mortality in STEMI patients with cardiogenic shock. Renal failure, PCI, and the requirement for inotropic or vasopressor drugs are also factors that influence in-hospital mortality.

**Supplementary Information:**

The online version contains supplementary material available at 10.1186/s43044-024-00599-8.

## Background

ST elevation myocardial infarction (STEMI) remains the world’s leading cause of death in patients with acute myocardial infarction (AMI). Patients presenting with STEMI are over three times more likely to present with cardiogenic shock than those presenting with non-STEMI (8.6% of patients vs. 2.5% of patients) [1]. STEMI with cardiogenic shock continues to be the leading cause of death over the past two decades [2]. In many patients with AMI, there is still a significant chance of cardiogenic shock with a 50% mortality rate, even for patients with STEMI who immediately receive interventions [3].

Ventricular systolic dysfunction is the most common cause of AMI-related cardiogenic shock [4]. Studies have been conducted on left ventricular systolic dysfunction (LVSD) and right ventricular systolic dysfunction (RVSD), with LVSD having a significantly higher incidence than RVSD in cases of cardiogenic shock due to AMI (94.7% vs 5.3%), but the mortality rate of both is similar [5]. We postulate that simultaneous biventricular dysfunction (BVD) results in a more pronounced hemodynamic compromise than unilateral dysfunction and becomes the strongest predictor for patient mortality. However, studies related to BVD are rarely reported [6]. It is known that the ventricular function examination is very easy to perform, both with left ventricular ejection fraction (LVEF) as a parameter of the left ventricle (LV) systolic function and tricuspid annulus systolic excursion (TAPSE) as a parameter of the right ventricle (RV) systolic function so that it can describe the condition of ventricular systolic function quickly [7].

Various classifications of cardiogenic shock have been developed to improve STEMI patient management to reduce mortality from cardiogenic shock [8]. The most recent is the classification of cardiogenic shock based on The Society for Cardiovascular Angiography and Intervention (SCAI), which was created to detect the presence of cardiogenic shock as early as possible to reduce mortality. Based on the SCAI classification, cardiogenic shock is divided into five stages, namely: At risk (A), Beginning (B), Classic (C), Deteriorating/doom (D), and Extremis (E), which shows a progressively higher mortality rate with each stage. With its timely application, a more accurate classification can be followed by appropriate interventions, and better outcomes in preventing mortality [9, 10]. However, the SCAI staging system does not incorporate various forms of ventricular dysfunction in the assessment of prognosis, especially in patients with biventricular dysfunction, theoretically a more significant predictor of mortality. Accordingly, we aimed to investigate the role of BVD in STEMI patients with cardiogenic shock to determine whether this echocardiographic assessment has additional prognostic utility.

## Methods

### Data population

**Fig. 1:**
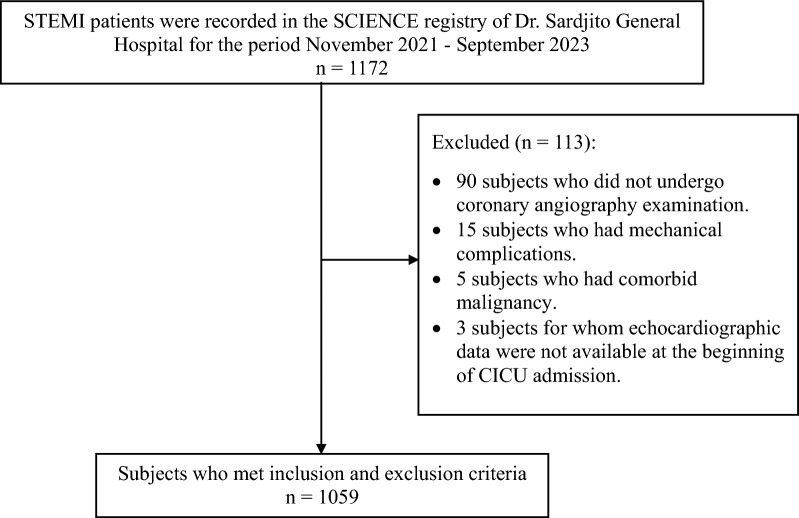
Flow diagram showing study inclusion and exclusion criteria. Patients with STEMI from November 2021 to September 2023 were included. The total number of eligible subjects in this study were 1059. *STEMI* ST elevation myocardial infarction; *SCIENCE* Sardjito Cardiovascular Intensive Care

Subjects were STEMI patients with cardiogenic shock admitted to the cardiac intensive care unit (CICU) at Dr. Sardjito General Hospital from November 2021 to September 2023 and registered to the Sardjito Cardiovascular Intensive Care (SCIENCE) registry. Inclusion criteria consisted of the following: (1) Age over 18 years; (2) Patients with a diagnosis of STEMI according to the diagnosis criteria in the 2017 European Society of Cardiology (ESC) Guidelines for the management of AMI in patients presenting with ST segment elevation. Exclusion criteria were: (1) Patients with comorbid malignancy; (2) Patients experiencing mechanical complications (ventricle septal rupture or acute mitral regurgitation); (3) Patients without echocardiography data of LV and RV function; and (4) Patients who did not undergo coronary angiography. From this SCIENCE registry, we selected data from 1,059 patients who met the inclusion and exclusion criteria for analysis (Fig. 1).

### Data collection

Patient demographics were recorded with vital signs, laboratory, clinical outcome data, procedures, and therapies performed during the CICU and hospital stay. The admission value of all critical signs, clinical measurements, and laboratory values was defined as the first value recorded after or closest to CICU admission. Transthoracic echocardiography (TTE) was done within one day after CICU admission with available data for LVEF and TAPSE. Admission diagnosis and management during treatment were defined as all diagnostics and management assessed within one day of CICU admission (supplementary data Table 1).

### Echocardiographic data

All patients underwent echocardiographic examination with a VIVID S6 or VIVID T8 machine (General Electric, USA) by a physician according to the European Association of Cardiovascular Imaging/American Society of Echocardiography guidelines. The 2D echocardiography data were sent to a computer workstation for further online analysis. Kappa calculations were made to assess the consistency of interobserver measurements. The interobserver variation value was 80% [11]. The LVSD in this study was determined if the ejection fraction (EF) was in the moderate or severe category, i.e., LVEF ≤ 40%, measured by the Simpson method. The Simpson biplane method uses manual tracing of the endocardium in the LV cavity during end-systole and diastole phase conditions from the apical four-chamber and two-chamber views. The RVSD in this study was determined if TAPSE was < 17 mm on apical four-chamber echocardiographic examination with M-mode [7]. Biventricular dysfunction (BVD) was identified when there were both left and right ventricular systolic dysfunction.

### Cardiogenic shock due to STEMI

Cardiogenic shock due to STEMI is defined as a condition in which the patient experiences cardiogenic shock based on the SCAI cardiogenic shock classification which is based on signs of hypotension (BP or MAP), hypoperfusion (pH or lactate levels), and administration of supportive therapy (vasoactive drugs, inotropic drugs, or mechanical circulatory support). The diagnosis was made at the time of treatment in the CICU. The SCAI classification of cardiogenic shock was the last recorded SCAI classification when the condition was diagnosed and was expressed as levels A, B, C, D, or E [10].

### Mortality

Mortality included all-cause mortality that occurred during hospitalization, both during treatment in the CICU and in the ward.

### Statistical analysis

The statistical analysis was conducted with SPSS software version 23 (IBM Corp., Armonk, NY) with a Windows operating system. Beginning with a descriptive analysis of the essential characteristics of the research subject, the numerical data were presented as mean ± SD and the categorical research data with frequency and percentage. The bivariate analysis used chi-square or Fisher exact tests for categorical data and Student T tests for numerical data. Logistic regression was used to determine the association between variables of interest and hospital mortality. The predictors with *p* value < 0.25 were considered to run a multivariate analysis with logistic regression. The *p* value < 0.05 was considered statistically significant. Then, we conducted a correlation matrix assessing the correlation coefficients between several variables to identify the strength and direction of the relationship between variables. Correlation coefficient above 0.8 can indicate multicollinearity.

## Results

### Patient characteristics

The mean ± SD age of the population was 59 ± 11 years, and 80.5% were man. There were 101 subjects (9.5%) who had a history of coronary artery disease (CAD), with 10 subjects who died (9.9%) and 91 subjects (90.1%) who survived. There were 44 subjects (4.2%) who had a history of heart failure (HF), with nine subjects (20.5%) who died and 35 subjects (79.5%) survived. There were 281 subjects (26.5%) who had a history of diabetes mellitus (DM), with 58 subjects (20.6%) who died and 223 subjects (79.4%) who survived. From 121 subjects (11.4%) with a history of stroke or acute stroke, there were 28 subjects (23.1%) who died, and 93 subjects (76.9%) who survived. There were 436 subjects (41.2%) with renal failure, with 137 subjects (31.4%) who died and 299 subjects (68.6%) who survived. The incidence of multivessel disease (MVD) in this study showed that of the 638 subjects (60.2%) with MVD, 116 subjects died (18.2%), and 521 subjects (81.8%) survived. There were 260 subjects (24.6%) with malignant arrhythmia, with 69 subjects (26.5%) who died and 191 subjects (73.5%) who survived. There were 37 subjects (28.2%) who died out of a total of 131 (12.4%) subjects with infection. There were 78 subjects (14.6%) who died by nonanterior AMI, 86 subjects (17%) who died by anterior AMI, and there was one subject (5%) who died from both anterior and nonanterior AMI (Table 1).

**Table 1:** Baseline characteristics

Variable	Overall, *n* = 1059	Mortality (%)		*p*
		Yes	No	
		165 (15.6)	894 (84.4)	
Age (years)	59 ± 11	62 ± 12	59 ± 11	< 0.001
Age > 75 Years, n (%)	84 (7.9)	19 (22.9)	64 (77.1)	0.060
Male	853 (80.5)	128 (15)	725 (85)	0.346
*Comorbidities*				
History of CAD, n (%)	101 (9.5)	10 (9.9)	91 (90.1)	0.131
History of HF, n (%)	44 (4.2)	9 (20.5)	35 (79.5)	0.485
DM, n (%)	281 (26.5)	58 (20.6)	223 (79.4)	0.008*
Stroke/ history of Stroke, n (%)	121 (11.4)	28 (23.1)	93 (76.9)	0.021*
Renal failure, n (%)	436 (41.2)	137 (31.4)	299 (68.6)	< 0.001*
MVD, n (%)	638 (60.2)	116 (18.2)	521 (81.8)	0.005*
Malignant arrhythmia, n (%)	260 (24.6)	69 (26.5)	191 (73.5)	< 0.001*
Infection, n (%)	131 (12.4)	37 (28.2)	94 (71.8)	< 0.001*
*Infarct location, n (%)*				0.219
Nonanterior	533 (50.3)	78 (14.6)	455 (85.4)	
Anterior	505 (47.7)	86 (17)	419 (83)	
Anterior and Nonanterior	21 (2)	1 (5)	20 (95)	
*Vital sign*				
Systolic BP (mmHg)	119 ± 22	107 ± 22	122 ± 21	< 0.001*
Diastolic BP (mmHg)	70 ± 13	63 ± 13	71 ± 13	< 0.001*
MAP (mmHg)	86 ± 15	77 ± 15	88 ± 14	< 0.001*
Heart rate (beat/minute)	84 ± 20	100 ± 23	82 ± 19	< 0.001*
*Laboratory finding*				
Creatinine (mg/dL)	1.49 ± 1.01	2.42 ± 179	1.32 ± 0.75	< 0.001*
GFR (ml/min/1,73m^2^.)	66 ± 29	41 ± 24	71 ± 27	< 0.001*
pH (mEq/L) (431 patients)	7.37 ± 0.09	7.34 ± 0.11	7.39 ± 0.07	< 0.001*
Lactate (mmol/L) (421 patient)	3.28 ± 2.7	4.73 ± 3.47	2.58 ± 1.91	< 0.001*
*Hemodynamic echocardiography*				
LVEF (%)	46 ± 13	38 ± 13	47 ± 12	< 0.001*
TAPSE (mm)	18 ± 4	16 ± 5	18 ± 4	< 0.001*
CO (L/minute) (1012 patients)	3.60 ± 1.09	3.52 ± 1.39	3.61 ± 1.03	0.468
CI (L/minute/m^2^) (1012 patients)	2.14 ± 0.68	2.11 ± 0.85	2.14 ± 0.64	0.703
SVR (dynes-sec/cm^−5^) (1004 patient)	1790 ± 639	1619 ± 630	1819 ± 636	< 0.001*
BVD	151 (14.3)	48 (31.8)	103 (68.2)	< 0.001*
*Management*				
Inotropic*/* vasopressor, n (%)	283 (26.7)	119 (42)	164 (58)	< 0.001*
IABP, n (%)	5 (0.5)	3 (60)	2 (40)	0.029*
PCI, n (%)	991 (93.6)	146 (14.7)	845 (85.3)	0.006*
*SCAI classification*				N/A
A	684 (64.6)	27 (3.9)	657 (96.1)	
B	83 (7.8)	17 (20.5)	66 (79.5)	
C	163 (15.4)	41 (25.2)	122 (74.8)	
D	106 (10)	62 (58.5)	44 (41.5)	
E	23 (2.2)	18 (78.3)	5 (21.7)	

*BP* blood pressure; *MAP* mean arterial pressure; *CAD* coronary artery disease; *DM* diabetes mellitus; *MVD* multivessel disease; *GFR* glomerular filtration rate; *LVEF* left ventricle ejection fraction; *TAPSE* tricuspid annular plane systolic excursion; *CO* cardiac output; *CI* cardiac index; *SVR* systemic vascular resistance; *BVD* biventricular dysfunction; *IABP*: intra-aortic balloon pump; *SCAI* the society for cardiovascular angiography and intervention; *PCI* percutaneous coronary intervention; *N/A* not assessed

Data represented as mean ± standard deviation for continuous variables and n (%) for categorical variables. * Significant *p* value < 0.05 (chi-square test or Fisher exact tests for categorical data and independent t-tests for numerical data)

The cardiac systolic function profile showed that in subjects with BVD, there were 48 subjects (31.8%) who died out of a total of 151 subjects. There were 283 subjects (26.7%) who received inotropic/vasopressor therapy with 119 subjects (42%) who died. An intra-aortic balloon pump (IABP) was implanted in five subjects (0.5%), with three subjects (60%) who died. Data from the subjects who underwent percutaneous coronary intervention (PCI) showed that there were 146 subjects (14.7%) who died out of 991 subjects who underwent PCI. Based on the classification of SCAI A, B, C, D, and E, 27 subjects (3.9%) died in the category of SCAI A. In SCAI B, 17 subjects (20.5%) died. In SCAI C, 41 subjects (25.2%) died. In SCAI D, 62 subjects (58.5%) died. The classification SCAI E showed 18 subjects (78.3%) who died (Table 1).

This study also adjusted for variables that had a *p* value < 0.25 for multivariate logistic regression. Table 2 shows the results of multivariate logistic regression analysis indicating BVD was significantly associated with in-hospital mortality with an odds ratio (OR) 1.771 (95% CI 1.771–2.819; *p* = 0.016). The results showed that the risk of mortality events during treatment increased 1.771 times in subjects with BVD. Several other variables also have a joint influence on mortality events during treatment with a *p* < 0.05 value, namely renal failure, PCI, and inotropic/vasopressor drugs.

**Table 2:** Multivariate logistic regression analysis of in-hospital mortality outcomes

Variable	*p*	OR	95% CI
BVD	0.016*	1.771	1.113–2.819
Renal failure	< 0.001*	5.122	3.233–8.116
PCI	0.044*	0.493	0.248–0.981
Inotropic/Vasopressor	< 0.001*	6.876	4.583–10.315

*CI* confidence interval; *OR* odds ratio; *BVD* biventricular dysfunction; *PCI* percutaneous coronary intervention

* Significant *p* value < 0.05 (logistic regression test)

In this study, we found four variables affecting mortality, so we conducted a correlation matrix analysis. Based on the data in Table 3, the correlation coefficients are all very close to zero, which indicates a weak or negligible linear relationship between the variables. This suggests that the variables do not influence each other in this dataset. Weak correlations imply that the presence of one condition or treatment does not strongly predict the presence of another condition or treatment. In clinical practice, a weak correlation implies that the presence of one condition or treatment does not strongly predict the presence of another condition or treatment. This may indicate that these factors act relatively independently in influencing patient outcomes.

**Table 3:** Correlation matrix

	BVD	Renal Failure	PCI	Inotropic/vasopressor
BVD	1.000	− 0.077	0.056	− 0.041
Renal Failure	− 0.077	1.000	0.020	− 0.188
PCI	0.056	0.020	1.000	− 0.073
Inotropic/vasopressor	− 0.041	− 0.188	− 0.073	1.000

A correlation coefficient above 0.8 can indicate multicollinearity

*BVD* biventricular dysfunction; *PCI* percutaneous coronary intervention

### Biventricular dysfunction vs. left or right ventricular systolic dysfunction

This study further subanalyzed the effect of each ventricular systolic dysfunction on in-hospital mortality outcomes. Based on the analysis, BVD showed the highest risk of mortality events compared to isolated LVSD or RVSD with RR 2.467 (95% CI 1.849–3.292; *p* < 0.001) and isolated LVSD with RR 1.847 (95% CI 1.381–2.470; *p* < 0.001). In contrast, this study found that isolated RVSD was not significantly associated with in-hospital mortality with RR 1.276 (95% CI 0.914–1.783; *p* = 0.193) (Table 4).

**Table 4:** Effect of each ventricular systolic dysfunction on in-hospital mortality outcomes

Variable	Mortality, n%		*p*	RR	95% CI
	Yes	No			
BVD	48 (31.8)	103 (68.2)	< 0.001*	2.467	1.849–3.292
Without BVD	117 (12.9)	791 (87.1)			
Isolated LVSD	53 (24.5)	163 (75.5)	< 0.001*	1.847	1.381–2.470
Without isolated LVSD	112 (13.3)	731 (86.7)			
Isolated RVSD	36 (18.9)	154 (81.1)	0.193	1.276	0.914–1.783
Without isolated RVSD	129 (14.8)	740 (85.2)			

*CI* confidence interval; *RR* relative risk; *BVD* biventricular dysfunction; *LVSD* left ventricular systolic dysfunction; *RVSD* right ventricular systolic dysfunction

* Significant *p* value < 0.05 (chi-square test)

### Number of mortality events and ventricular systolic dysfunction at each SCAI shock stage

**Fig. 2:**
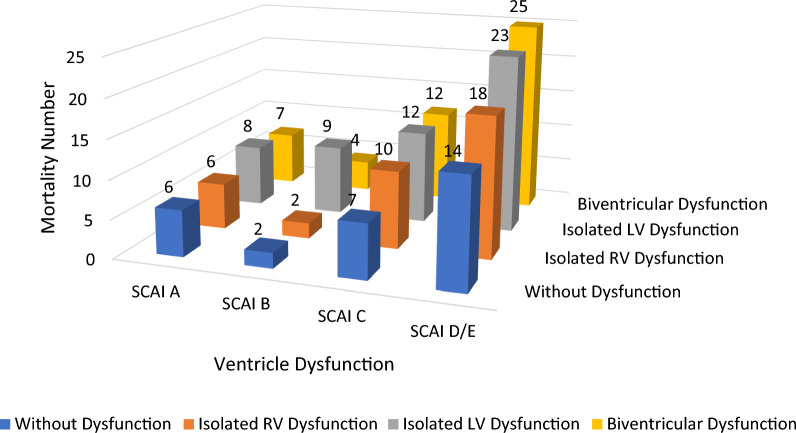
Three-dimensional bar graph showing hospital mortality, ventricular systolic dysfunction, and SCAI shock stage. *LV* left ventricular; *RV* right ventricular; *SCAI* Society for Cardiovascular Angiography and Interventions. Because of the low number of patients in SCAI shock stage E, we grouped SCAI shock stages D and E for analysis

The distribution of patients with ventricular systolic dysfunction in this study showed some variation in the incidence of ventricular systolic dysfunction at each SCAI level. Figure 2 shows that the more severe the degree of SCAI, the higher the incidence of ventricular dysfunction. SCAI C and D/E show the highest number of ventricular dysfunction events, while SCAI C and D/E represent moderate to severe cardiogenic shock. In contrast, SCAI A and B are the initial conditions of mild cardiogenic shock, so the incidence of ventricular systolic dysfunction is relatively low.

## Discussion

This is the first study in Indonesia to assess the effect of ventricular systolic function on in-hospital mortality in STEMI patients with cardiogenic shock. Research has shown that in patients who experience STEMI, the incidence of ventricular systolic dysfunction is the most common condition, and it increases the mortality risk of STEMI patients who experience cardiogenic shock [12]. This study demonstrates that BVD significantly increases the risk of in-hospital mortality in STEMI patients with cardiogenic shock, even when compared with isolated LVSD or RVSD. Consistent with research by Burstein et al. [6], patients with BVD conditions have a 1.670 times higher risk of mortality (95% CI 1.220–2.286; *p* = 0.001). However, in that study, the research subjects involved all patients with cardiogenic shock of any cause.

The pathophysiological mechanisms underlying BVD in STEMI patients with cardiogenic shock become the stronger predictive value for mortality because BVD leads to reduced cardiac output, causing systemic hypoperfusion and increased intracardiac pressures [13]. The combination of left and right ventricular failure results in more severe heart failure symptoms compared to isolated dysfunction. Biventricular dysfunction (BVD) likely exacerbates hemodynamic instability more than isolated LVSD or RVSD by impairing both cardiac output and venous return, leading to more severe heart failure symptoms and higher mortality risk [14]. Moreover, the occurrence of BVD incites considerable neurohormonal activation, which may exacerbate cardiac performance and elevate the risk of mortality [15]. Inflammatory responses are also amplified, contributing to further hemodynamic compromise [13].

The findings of this study have important clinical implications, as they highlight the critical importance of early recognition and management of BVD in STEMI patients with cardiogenic shock. Prompt diagnosis, expedient stabilization, and appropriate triage and consultation can drastically alter the course of cardiogenic shock [16]. The use of bedside echocardiography in the CICU department allows for rapid assessment of ventricular function and can aid in the examination that can provide powerful hemodynamic data on cardiac function, which can inform the management of patients in the CICU department without the need for invasive hemodynamic monitoring [17]. This study further emphasizes that bedside echocardiography is useful in risk-stratifying STEMI patients with mortality factors associated with cardiogenic shock at the CICU department. Furthermore, the availability of mechanical circulatory support, such as short-term percutaneous left ventricular assist devices, can be critical for patients with refractory shock, providing temporary support while addressing underlying issues [18].

Another condition that significantly influences in-hospital mortality in cardiogenic shock patients is renal failure. Early detection and management of renal function are crucial because the high mortality risk associated with renal failure highlights the need for aggressive management strategies to support renal function and mitigate its impact on overall patient outcomes. Acute kidney injury (AKI) is common in CS patients, with studies showing a mortality rate of 63% in those with AKI compared to 36% without AKI [19]. Similarly, this study shows that STEMI patients with cardiogenic shock who experience renal failure will have an increased risk of in-hospital mortality. This study also showed similar results to a previous study conducted by Bagaswoto et al. [20], with the research population of patients admitted to the CICU of Dr. Sardjito General Hospital (OR, 2.27; 95% CI 1.06–4.28; *p* = 0.035). Another study also reported that in patients with cardiogenic shock caused by STEMI with renal failure, there was an increased risk of in-hospital mortality, and renal failure was the main predictor of mortality (RR, 17.0; 95% CI 2.5–117.1; *p* = 0.001) [21]. Several approaches to the management of renal failure have been implemented, such as renal replacement therapy; however, mortality during treatment remains high, indicating the need for improved therapeutic interventions and early detection strategies. This condition can worsen the incidence of cardiogenic shock [22].

The management of STEMI with PCI has become well-established and recommended as the first-choice therapy, with significant mortality reduction benefits [23]. Additionally, based on the ESC STEMI guidelines, STEMI patients with cardiogenic shock presentation are recommended for primary PCI action [12]. Similarly, this study shows PCI can reduce in-hospital mortality. It has long been reported that STEMI patients with cardiogenic shock who underwent early PCI had better outcomes, as seen in the SHOCK study that showed the survival rate in 30 days of successful post-PCI patients was 65% versus 20% of those who were unsuccessful [24].

Our study showed that patients with cardiogenic shock who requiring for inotropic or vasopressor support are strongly associated with higher in-hospital mortality. This finding indicates the severity of the clinical condition in patients requiring such support. The necessity for inotropic or vasopressor support indicates significant hemodynamic instability and unfavorable prognosis. Close monitoring and management are critical for patients requiring such support to enhance survival prospects. Additionally, the selection of inotropic and vasopressor medications markedly affects patient outcomes, given their diverse risk–benefit profiles. For example, dobutamine is employed for augmenting myocardial contractility, yet it is linked to heightened arrhythmias and increased oxygen demand, which may worsen mortality rates [25]. In contrast, noradrenaline seems to present a more advantageous safety profile, especially when utilized in conjunction with levosimendan, which may enhance cardiac output while not significantly elevating risk. This highlights the importance of personalized treatment strategies based on individual hemodynamic profiles, emphasizing that optimizing pharmacological interventions could be pivotal in enhancing survival rates among this vulnerable population [26].

This study was a single-center study, which certainly has some limitations. Despite its large sample size and detailed data, the study has several limitations inherent to all retrospective cohort studies, including the need for prospective validation so that the potential presence of unmeasured confounding factors and missing data that may affect the results are avoided like variations in treatment protocols, differences in patient selection, time-to-treatment, or duration of shock that may impact outcomes. Also, our center at Dr. Sardjito General Hospital in Yogyakarta, Indonesia, is a tertiary care referral CICU in the Javanese area that tends to be unique in terms of demographics, characteristics living culture, and logistics compared to other areas and institutions so prospective multicenter study would be needed to validate the results of this study.

## Conclusion

Biventricular dysfunction significantly increases the risk of in-hospital mortality among patients presenting with ST elevation myocardial infarction (STEMI) with cardiogenic shock. Additionally, renal impairment, percutaneous coronary intervention (PCI), and the necessity for inotropic or vasopressor drugs are further determinants that affect in-hospital mortality rates.

## Supplementary Information

Additional file 1.

## Data Availability

The data generated in this study are available from the corresponding author upon reasonable request.

## Abbreviations

STEMI: ST elevation myocardial infarction
AMI: Acute myocardial infarction
LVSD: Left ventricular systolic dysfunction
RVSD: Right ventricular systolic dysfunction
BVD: Biventricular dysfunction
LVEF: Left ventricle ejection fraction
LV: Left ventricle
RV: Right ventricle
TAPSE: Tricuspid annular plane systolic excursion
SCAI: The society for cardiovascular angiography and intervention
CICU: Cardiac intensive care unit
SCIENCE: Sardjito cardiovascular intensive care
TTE: Transthoracic echocardiography
EF: Ejection fraction
CAD: Coronary artery disease
HF: Heart failure
DM: Diabetes mellitus
MVD: Multivessel disease
PCI: Percutaneous coronary intervention.
RR: Relative risk
CI: Confidence interval
OR: Odds ratio
VT: Ventricular tachycardia
TAVB: Total atrioventricular block
SIRS: Systemic inflammatory response syndrome
AKI: Acute kidney injury
