# Supplementary material for: Biventricular dysfunction predicts mortality in ST elevation myocardial infarction patients with cardiogenic shock
Source: Egypt Heart J. 2025 Jan 8;77:7. doi: 10.1186/s43044-024-00599-8 (PMC11711729; doi:10.1186/s43044-024-00599-8)
Supplement: Supplementary file 1 — Additional file 1. [file 43044_2024_599_MOESM1_ESM.docx]

Supplementary **table 1.** Definitions of diagnosis and management of the study.

| **Term** | **Definition** |
| --- | --- |
| History of CAD | History of coronary artery disease (CAD) before hospitalisation is a patient with a history of previous acute coronary syndrome (ACS) or a history of previous reperfusion treatment using either fibrinolytic strategies, percutaneous coronary intervention (PCI), or coronary artery bypass surgery or known to have significant CAD from previous invasive coronary angiography or computerized tomography-angiography examinations, having been admitted to the intensive care unit (ICU) due to a heart attack, obtained from the patient's autoanamnesis or heteroanamnesis or based on patient medical records. |
| History of HF | History of heart failure (HF) is if the patient has a history of chronic heart failure (CHF) before admission. Heart failure is defined as the presence of clinical findings of HF in the presence of complaints of dyspnea on exertion, dyspnea in the supine position (orthopnea), fluid retention or the presence of rales, jugular venous distension, and pulmonary oedema. Classification of HF based on ejection fraction: Heart failure with reduced ejection fraction (HFrEF) is CHF with an ejection fraction ≤ 40%. Heart failure with mildly reduced ejection fraction (HFmEF), which is CHF with an ejection fraction of 41-49%. Heart failure with preserved ejection fraction (HFpEF) is CHF with ejection fraction >50%, or based on patient medical records [27]. |
| DM | Diabetes mellitus (DM) is a metabolic disease characterised by hyperglycemia with fasting plasma glucose ≥ 126 mg/dl or plasma glucose ≥ 200 mg/dl 2 hours after an oral glucose tolerance test with a glucose load of 75 g or current plasma glucose ≥ 200 mg/dl with classic complaints or HbA1c examination ≥ 6.5%, or based on records of DM history and/or DM treatment in the patient's medical record [28]. |
| Stroke/history of stroke | A stroke or history of stroke is a patient diagnosed with/or has a history of cerebrovascular disease, which includes ischemic stroke, hemorrhagic stroke, or transient ischemic attack based on the patient's history, physical examination, or medical records [29]. |
| Renal failure | Renal failure is when the patient is diagnosed with chronic kidney disease (CKD), acute renal insufficiency, or worsening renal function as evidenced by the examination of the glomerular filtration rate (GFR) <60 mL/min/1.73 m^2^ based on the 2021 CKD-EPI formula or based on the patient's medical records [30]. |
| Infarct Location | Infarct location is the location of myocardial infarction determined based on electrocardiographic recordings when the diagnosis of STEMI was made. Infarct location is divided into three groups, namely anterior, nonanterior (inferior, dextral, posterior, high lateral), and both anterior and nonanterior. |
| MVD | The multivessel disease (MVD) is ≥70% luminal stenosis in ≥ 2 main coronary artery branches or one main coronary artery branch with 50% stenosis in the left main coronary artery (LMCA), obtained on diagnostic angiography of PCI procedure [31]. |
| Malignant arrhythmia | Malignant arrhythmia is the presence of ventricular tachycardia (VT), ventricular fibrillation (VF), or total atrioventricular block (TAVB) during treatment, as proven by a 12-lead electrocardiogram (ECG) or monitor during treatment [32, 33]. |
| Infection | Infection is the presence of infectious comorbidity (pneumonia, urinary tract infection, or other infection) based on the examination performed. Once referred to an internal medicine colleague, it is approved as an infection when the diagnosis of STEMI is confirmed or based on the history of medical records [34]. |
| Inotropic/vasopressor | Vasopressor/inotropic therapy is a pharmacological therapy given to the patient to increase the patient's blood pressure assessed at the time of initial admission to the intensive cardiac care unit (ICCU). |
| IABP | Intra-aortic balloon pump (IABP) therapy was defined as the condition of the research subjects who underwent treatment with IABP installation at Dr. Sardjito General Hospital, Yogyakarta, Indonesia. |
